# Editorial: Calcium regulation and Mechano-transduction in vascular diseases: obligatory role of transient receptor potential and Piezo channels

**DOI:** 10.3389/fphys.2024.1375432

**Published:** 2024-02-28

**Authors:** Ramon J. Ayon, Maniselvan Kuppusamy, Giulio Ceolotto, Yen-Lin Chen

**Affiliations:** ^1^ Department of Molecular Physiology and Biological Physics, School of Medicine, University of Virginia, Charlottesville, VA, United States; ^2^ Robert M. Berne Cardiovascular Research Center, School of Medicine, University of Virginia, Charlottesville, VA, United States; ^3^ Department of Medicine-DIMED, University of Padua, Padua, Italy

**Keywords:** mechanotranduction, calcium signaling, piezo 1, TRP channel, vascular remodeling, fibroblast, endothelail cell, smooth musle cell

Maintaining vascular tone is essential to ensuring that our body’s tissues receive the necessary oxygen and nutrients to meet their metabolic demands. The cells responsible for this regulation are vascular cells, which include endothelial cells (EC) and smooth muscle cells (SMC). Together, they control the diameter of the blood vessel lumen to ensure optimal blood flow throughout the body. The improper regulation of vascular tone can have significant negative impacts on the body’s overall health. Dysfunctional regulation of blood vessels can result in elevated blood pressure, commonly known as hypertension. This condition is a significant risk factor for cardiovascular diseases such as heart failure, and if left untreated, it can lead to serious complications such as heart attack or stroke.

Vascular cells can sense external transmembrane forces, such as pressure, stretch, and shear stress, and respond by converting this stimulus into electrochemical signals, a process known as mechanotransduction. Piezo1 was one of the first discovered mechanosensitive ion channels that would respond to perturbations across the membrane. In response to this stimulus, the open probability of Piezo1 increases, allowing ions such as calcium to enter the cell and triggering secondary messenger systems that influence a variety of biological functions, including vascular development and blood pressure regulation (Li et al., 2014; Beech and Kalli, 2019; Lai et al., 2021). Piezo1 channels have been implicated in the stimulation of NO production and NO-dependent vasodilation in mouse mesenteric (Wang et al., 2016), pulmonary (Lhomme et al., 2019), and uterine (John et al., 2018) arteries. The expression and function of Piezo1 are also critical components in vascular development (Li et al., 2014; Ranade et al., 2014) and vascular smooth muscle remodeling (Retailleau et al., 2015). Pathologically, Piezo1 signaling has been linked to various pathological processes, including chronic inflammation, fibrosis, and carcinogenesis. More recently, there have been intense studies on the mechanosensitive Piezo1 channels and their pathological role in cardiovascular disease.

The Frontiers Research Topic “*Calcium regulation and Mechano-transduction in vascular diseases: obligatory role of transient receptor potential and Piezo channels*” encompasses four articles highlighting various aspects of the mechanical forces sensed by vascular cells that are translated into intracellular Ca^2+^ signaling events that regulate vascular function. These aspects include Piezo1 channels and the regulation of vascular tone by altering vasoconstriction and vasorelaxation. The second focus is aligned with vascular remodeling, i.e., Piezo’s role in cardiac fibrosis and vascular calcification.

During pregnancy, the body undergoes cardiovascular changes to meet the metabolic demands of both the mother and the fetus. As a result, there is an increase in uterine arterial blood flow. However, despite the increase in blood flow, there is no change in blood pressure. This is because the uterine arteries (UA) dilate to accommodate the greater blood flow. The Piezo1 channel, which is highly expressed in endothelial cells (EC) and smooth muscle cells (SMCs) of resistance arteries such as UAs, is responsible for sensing mechanical forces. In this research article, Arishe et al. sought to examine the underlying mechanisms of Piezo1 signaling in the UAs of pseudo-pregnant rats. They found that concentration-dependent relaxation to the Piezo1 agonist Yoda1 was greater in UAs from pseudo-pregnant rats when compared to virgin rats. Furthermore, they discovered that the nitric oxide synthase inhibitor L-NAME was able to attenuate Yoda1-induced relaxation response in UA. Thus, Arishe et al. were able to demonstrate that Piezo1 channels regulate vasodilation in the UAs of pseudo-pregnant rats in part via a NO-dependent mechanism.

Pulmonary arterial hypertension (PAH) is a life-threatening condition characterized by increased pulmonary arterial pressure (PAP), which is caused by elevated pulmonary vascular resistance (PVR) (Balistrieri et al., 2023). Sustained pulmonary vasoconstriction, concentric vascular remodeling, and other factors are major causes of direct increases in PVR and PAP in patients with PAH. Current therapeutic targets for pulmonary arterial hypertension (PAH) focus on membrane receptors and ion channels that promote vasodilation while inhibiting vasoconstriction and proliferation (Spiekerkoetter et al., 2019). Calcium channel blockers are frequently used to relieve pulmonary vasoconstriction. However, the treatment proves effective for only a small number of patients, and many of them eventually develop resistance to it. The TRP family of ion channels plays a crucial role in store-operated calcium entry in the pulmonary circulation and has been studied extensively as a potential target for therapeutic intervention. However, targeting TRP channels therapeutically is a challenging task due to their pharmacological non-specificity and the potential implications for unintended effects on other tissues (Koivisto et al., 2022). Yang et al. propose Piezo1 as a therapeutic target for treating pulmonary hypertension. Yang et al. There is evidence of increased Piezo1 expression in the PASMCs and PAECs of patients with PAH, suggesting Piezo1’s pathological implication in PAH. The authors have brought up the discussion of several newly discovered Piezo1-targeting compounds. These compounds could prove to be promising in the clinical setting. The compound Salvianolic acid B (SalB), extracted from Chinese sage root, was shown to inhibit Piezo1 ion channels activated by Yoda1 or mechanical stress (Pan et al., 2022). The triterpenoid saponin Tubeimoside I (TBMS I) has also been demonstrated to antagonize Yoda1-induced Piezo1 channel activation (Liu et al., 2020). These compounds are expected to become valuable pharmacological tools for examining the function of the Piezo1 channel. We hope that they can be utilized to further investigate the pathophysiology of cardiovascular diseases so that we can have a better understanding of the impact of Piezo1 in vascular biology.

Mechanotransduction is an important process in the repair and regeneration of tissues. However, prolonged or excessive mechanical stress is a major cause of fibrosis. Several studies have shown that various ion channels contribute to the proliferation, contraction, and secretion of myofibroblasts. The review by Xing et al. summarizes the research progress made on the role of TRP channels, Piezo1, Ca^2+^ release-activated channels (CRAC), and others in myocardial fibrosis Xing et al. Some key findings covered in the review include the role of TRP channels in fibroblast differentiation by regulating intracellular Ca^2+^ levels. There is also evidence that CRAC channels may also contribute to cardiac remodeling under pathological conditions (Feng et al., 2019). Additionally, Piezo1-mediated mechanotransduction was shown to promote cardiac hypertrophy by impairing calcium homeostasis to activate calpain/calcineurin signaling (Zhang et al., 2021).

Vascular calcification is linked to various cardiovascular diseases. However, it is yet to be determined how Piezo1 contributes to this process. Szabo et al. have investigated the role of Piezo1 in human aortic smooth muscle cells. They found that HAoSMCs conditioned with culture media that promotes calcification showed higher calcium transients compared to the control group. Activation of Piezo1 channels with Yoda1 was observed to increase the rate of calcification in human aortic smooth muscle cells (HAoSMCs), while the Piezo1 inhibitor Dooku1 was found to inhibit the calcification process. The data from the study strongly indicate that Piezo1 plays a crucial role in arterial calcification.

The four articles presented in this special topic shed light on the significance of mechanotransduction in both normal and abnormal physiological conditions. The findings provide potential new avenues for treating cardiovascular disease through the development of diagnostic methods, therapeutic drugs, and clinical management strategies. However, further research is necessary to bridge the gaps in our understanding of the pathogenesis of Cardiovascular Disease and promote progress in this field.

## References

[B1] Balistrieri A. Makino A. Yuan J. X. (2023). Pathophysiology and pathogenic mechanisms of pulmonary hypertension: role of membrane receptors, ion channels, and Ca(2+) signaling. Physiol. Rev. 103 (3), 1827–1897. 10.1152/physrev.00030.2021 36422993 PMC10110735

[B2] Beech D. J. Kalli A. C. (2019). Force sensing by Piezo channels in cardiovascular health and disease. Arteriosclerosis, Thrombosis, Vasc. Biol. 39 (11), 2228–2239. 10.1161/ATVBAHA.119.313348 PMC6818984 31533470

[B13] Feng J. Armillei M. K. Yu A. S. Liang B. T. Runnels L. W. Yue L. (2019). Ca(2+) signaling in cardiac fibroblasts and fibrosis-associated heart diseases. J. Cardiovasc Dev. Dis. 6 (4), 34. 10.3390/jcdd6040034 31547577 PMC6956282

[B3] John L. Ko N. L. Gokin A. P. Gokina N. I. Mandalá M. Osol G. J. (2018). The Piezo1 cation channel mediates uterine artery shear stress mechanotransduction and vasodilation during rat pregnancy. Am. J. physiology Heart circulatory physiology 315 (4), H1019–H1026. 10.1152/ajpheart.00103.2018 PMC6230896 30004235

[B15] Koivisto A. P. Belvisi M. G. Gaudet R. Szallasi A. (2022). Advances in TRP channel drug discovery: from target validation to clinical studies. Nat. Rev. Drug Discov. 21, 41–59. 10.1038/s41573-021-00268-4 34526696 PMC8442523

[B4] Lai A. Cox C. D. Chandra Sekar N. Thurgood P. Jaworowski A. Peter K. (2021). Mechanosensing by Piezo1 and its implications for physiology and various pathologies. Biol. Rev. 97 (2), 604–614. 10.1111/brv.12814 34781417

[B5] Lhomme A. Gilbert G. Pele T. Deweirdt J. Henrion D. Baudrimont I. (2019). Stretch-activated Piezo1 channel in endothelial cells relaxes mouse intrapulmonary arteries. Am. J. Respir. Cell Mol. Biol. 60 6, 650–658. 10.1165/rcmb.2018-0197OC 30562052

[B6] Li J. Hou B. Tumova S. Muraki K. Bruns A. Ludlow M. J. (2014). Piezo1 integration of vascular architecture with physiological force. Nature 515 (7526), 279–282. 10.1038/nature13701 25119035 PMC4230887

[B7] Liu S. Pan X. Cheng W. Deng B. He Y. Zhang L. (2020). Tubeimoside I antagonizes yoda1-evoked Piezo1 channel activation. Front. Pharmacol. 11, 768. 10.3389/fphar.2020.00768 32523536 PMC7261832

[B8] Pan X. Wan R. Wang Y. Liu S. He Y. Deng B. (2022). Inhibition of chemically and mechanically activated Piezo1 channels as a mechanism for ameliorating atherosclerosis with salvianolic acid B. Br. J. Pharmacol. 179 (14), 3778–3814. 10.1111/bph.15826 35194776

[B9] Ranade S. S. Qiu Z. Z. Woo S. H. Hur S. S. Murthy S. E. Cahalan S. M. (2014). Piezo1, a mechanically activated ion channel, is required for vascular development in mice. Proc. Natl. Acad. Sci. U. S. A. 111 (28), 10347–10352. 10.1073/pnas.1409233111 24958852 PMC4104881

[B10] Retailleau K. Duprat F. Arhatte M. Ranade S. S. Peyronnet R. Martins J. R. (2015). Piezo1 in smooth muscle cells is involved in hypertension-dependent arterial remodeling. Cell Rep. 13 (6), 1161–1171. 10.1016/j.celrep.2015.09.072 26526998

[B14] Spiekerkoetter E. Kawut S. M. de Jesus Perez V. A. (2019). New and emerging therapies for pulmonary arterial hypertension. Annu. Rev. Med. 70, 45–59. 10.1146/annurev-med-041717-085955 30216732 PMC7735523

[B11] Wang S. P. Chennupati R. Kaur H. Iring A. Wettschureck N. Offermanns S. (2016). Endothelial cation channel PIEZO1 controls blood pressure by mediating flow-induced ATP release. J. Clin. Investigation 126 (12), 4527–4536. 10.1172/Jci87343 PMC5127677 27797339

[B12] Zhang Y. H. Su S. A. Li W. D. Ma Y. K. Shen J. Wang Y. P. (2021). Piezo1-Mediated mechanotransduction promotes cardiac hypertrophy by impairing calcium homeostasis to activate calpain/calcineurin signaling. Hypertension 78 (3), 647–660. 10.1161/Hypertensionaha.121.17177 34333987

